# Chemoreceptor Evolution in Hymenoptera and Its Implications for the Evolution of Eusociality

**DOI:** 10.1093/gbe/evv149

**Published:** 2015-08-12

**Authors:** Xiaofan Zhou, Antonis Rokas, Shelley L. Berger, Jürgen Liebig, Anandasankar Ray, Laurence J. Zwiebel

**Affiliations:** ^1^Department of Biological Sciences, Vanderbilt University; ^2^Department of Cell and Developmental Biology, University of Pennsylvania; ^3^Department of Genetics, University of Pennsylvania; ^4^Department of Biology, University of Pennsylvania; ^5^School of Life Sciences, Arizona State University, Tempe; ^6^Department of Entomology, University of California, Riverside

**Keywords:** chemosensation, odorant receptor, gustatory receptor, eusociality, Hymenoptera

## Abstract

Eusocial insects, mostly Hymenoptera, have evolved unique colonial lifestyles that rely on the perception of social context mainly through pheromones, and chemoreceptors are hypothesized to have played important adaptive roles in the evolution of sociality. However, because chemoreceptor repertoires have been characterized in few social insects and their solitary relatives, a comprehensive examination of this hypothesis has not been possible. Here, we annotate ∼3,000 odorant and gustatory receptors in recently sequenced Hymenoptera genomes and systematically compare >4,000 chemoreceptors from 13 hymenopterans, representing one solitary lineage (wasps) and three independently evolved eusocial lineages (ants and two bees). We observe a strong general tendency for chemoreceptors to expand in Hymenoptera, whereas the specifics of gene gains/losses are highly diverse between lineages. We also find more frequent positive selection on chemoreceptors in a facultative eusocial bee and in the common ancestor of ants compared with solitary wasps. Our results suggest that the frequent expansions of chemoreceptors have facilitated the transition to eusociality. Divergent expression patterns of odorant receptors between honeybee and ants further indicate differential roles of chemoreceptors in parallel trajectories of social evolution.

## Introduction

Chemosensation, or the perception of chemical cues (e.g. smell and taste) from biotic and abiotic sources, is fundamental to many aspects of insect lifecycles such as host-seeking (e.g. for blood-feeding mosquitoes), mating choice, and searching for oviposition sites (Suh et al. 2014). In social insects, where multiple individuals live as a group and cooperate on tasks like brood care and colony defense, chemosensation serves a particularly important role in mediating the recognition and communication between members of the same society (Wilson 1965). For instance, ants and other eusocial insects form sophisticated societies organized according to specialized behavioral castes, notably the reproductive caste (queen) and sterile caste (worker). The intricate interactions within and between castes that maintain the organization of such societies are coordinated via various types of signals such as chemical (e.g. queen pheromones) as well as acoustical and visual (e.g. the dance language in honeybee) cues (Seeley 1995; Slessor et al. 2005). Chemical communication is perhaps the most universal and important one of such mechanisms, and largely involves the accurate discrimination of a diverse set of cuticular hydrocarbons (CHCs) (Blomquist and Bagneres 2010). Therefore, it is reasonable to hypothesize that the sophisticated chemosensory systems in social insects, and their underlying molecular components, represent adaptations that have facilitated the evolution of sociality (LeBoeuf et al. 2013).

A key step in insect chemosensation is the detection of chemicals by receptor proteins present on peripheral sensory neuron membrane, which initiate downstream signaling ultimately leading to behavioral responses. Three classes of insect chemosensory receptors are currently known. Odorant receptors (Ors) and gustatory receptors (Grs) belong to the same superfamily and both have seven transmembrane domains. However they differ in their general functions: Ors are generally, but not exclusively, expressed in antenna and other chemosensory appendages where they detect various volatile compounds (Suh et al. 2014); in contrast, Grs display diverse expression patterns in gustatory organs and other tissues, and the few Grs with known function include receptors for sweet and bitter tastants, as well as for carbon dioxide (Suh et al. 2014). The third chemoreceptor class is comprised of ionotropic receptors (Irs), which are members of the ancient ionotropic glutamate receptor family (Benton et al. 2009). Their roles in chemosensation were only recently discovered and known ligands include amines and acids (Koh et al. 2014; Suh et al. 2014).

The genes encoding these chemoreceptors have experienced highly dynamic evolution in insects; they are among the most rapidly evolving genes in the genome (Neafsey et al. 2015), and their copy numbers can vary considerably among insect lineages (Sánchez-Gracia et al. 2011). Intriguingly, some of the largest chemoreceptor repertoires are found in eusocial insects. Each of the four ant genomes with available chemoreceptor annotations contains more than 300 *Ors* as well as 17–97 *Gr*s and 23–32 *Ir*s (Smith, Zimin, et al. 2011; Smith, Smith, et al. 2011; Zhou et al. 2012). The honeybee *Apis mellifera* also has 163 *Or*s (Robertson and Wanner 2006), which exceeds most solitary insects (Sánchez-Gracia et al. 2011). We recently compared the chemoreceptor repertoires of selected hymenopteran species and demonstrated that they are largely shaped by rapid gain-and-loss throughout the evolution of these lineages (Zhou et al. 2012). In particular, the *Or* family appears to have experienced a dramatic expansion in the common ancestor of ants (Zhou et al. 2012).

Although these findings are consistent with the adaptive role of chemoreceptors in the evolution of social insects, contradictory observations have also been made. For example, the solitary parasitoid jewel wasp *Nasonia vitripennis* contains ∼60 more *Or*s and ∼40 more *Gr*s than *A. mellifera* (Robertson et al. 2010), seemingly challenging the notion that social insects require a greater variety of chemoreceptors than solitary insects. Additionally, a recent molecular evolutionary comparison of *Ors* found lower levels of positive selection in ants relative to *N. vitripennis* (Roux et al. 2014). However, *N. vitripennis* is the only solitary hymenopteran species with high-quality chemoreceptor annotations. Therefore, it is difficult to determine whether *N. vitripennis* represents an exception in chemoreceptor evolution.

Recently, several additional hymenopteran genomes have become available, including: two solitary wasps, *Ceratosolen solmsi* (Xiao et al. 2013), and *Microplitis demolitor* (Burke et al. 2014); one socially polymorphic halictid bee, *Lasioglossum albipes* (Kocher et al. 2013), which represents another independent origin of eusociality in addition to honeybee and ants; and several additional ant species, notably the dorylomorph *Cerapachys biroi* which represents the second most early-branching ant species following the ponerine *Harpegnathos saltator* (Oxley et al. 2014; Schrader et al. 2014; Mikheyev and Linksvayer 2015). These new genomes provide powerful comparative genomics resources to study the evolutionary patterns of chemoreceptor genes in solitary and social insects. Here, we report characterization of the *Or* and *Gr* repertoires in eight hymenopteran genomes where careful chemoreceptor annotations were previously lacking. We focused on *Or* and *Gr* gene families because they showed more dramatic copy number variations among previously analyzed hymenopteran genomes (Zhou et al. 2012). By comparing >4,000 *Or*s and *Gr*s from 13 solitary and social Hymenoptera species, we further investigated the evolution of hymenopteran chemoreceptor genes at levels of copy number dynamics, sequence evolution, and expression divergence.

## Materials and Methods

### Gene Annotation

Genome assemblies of the eight hymenopteran species listed in table 1 were downloaded from their respective sources (supplementary table S8, Supplementary Material online). *Or* and *Gr* genes were annotated in these species as described previously (Zhou et al. 2012). In brief, protein sequences of previously reported insect *Or* and *Gr* genes were used as queries to perform TBLASTN (Nicholas et al. 1997) search (e value cutoff of 1e-5) against each of the eight hymenopteran genome. Putative *Or* and *Gr* coding regions were identified from the search results after filtering out low-scoring and short hits. For each putative coding region, the most similar query sequence was used as reference for homology-based gene prediction using GeneWise v2.2.0 (Birney et al. 2004). All predicted gene models were checked for the presence of the characteristic domains of Ors (IPR004117) or Grs (IPR009318 or IPR013604) in their coded protein sequences using InterProScan v5 (Jones et al. 2014). Previously annotated chemoreceptor genes of *N. vitripennis* (Robertson et al. 2010), *A. mellifera* (Robertson and Wanner 2006), and five other ants (Smith, Zimin, et al. 2011; Smith, Smith, et al. 2011; Zhou et al. 2012; Oxley et al. 2014) were used for this study. Genes encoding proteins no shorter than 350 amino acids were included in subsequent phylogenetic and selection analyses. It should be noted that *C. obscurior* and *M. pharaonis* have their genomes sequenced most recently during the preparation of this manuscript. The chemoreceptors of *C. obscurior* and *M. pharaonis* were annotated and provided as resources, but the phylogenetic and selection analyses were not rerun to include these two species because ants are already well represented in this study by eight other ant species.

**Table 1:** Numbers of *Or* and *Gr* Genes Annotated in This Study

Lineage	Species	*Or*		*Gr*	
		This Study^a^	Previous Report^b^	This Study^a^	Previous Report^b^
Solitary wasps	*Ceratosolen solmsi*	56 (1) [2]	44	5 (0) [1]	5
	*Microplitis demolitor*	203 (15) [4]	NA	79 (6) [1]	NA
Halictid bee	*Lasioglossum albipes*	158 (18) [8]	NA	23 (4) [0]	NA
Ants	*Acromyrmex echinatior*	375 (23) [57]	NA	116 (13) [34]	NA
	*Atta cephalotes*	341 (65) [89]	215	89 (14) [82]	25
	*Cardiocondyla obscurior*	232 (33) [7]	NA	34 (3) [1]	NA
	*Monomorium pharaonis*	240 (97) [31]	NA	159 (29) [17]	NA
	*Solenopsis invicta*	333 (86) [66]	297	219 (56) [60]	NA

^a^The figures indicate the number of chemoreceptor genes encoding proteins of at least 350 amino acids; the number of incomplete genes encoding proteins between 200 and 349 amino acids (in the parenthesis); the number of pseudogene are given (in the bracket).

^b^The numbers of previously reported chemoreceptor genes are from the following sources: *C. solmsi*—(Xiao, et al. 2013); *A. cephalotes*—(Koch, et al. 2013); and *S. invicta* (only *Or*s)—(Wurm, et al. 2011). “NA” denotes there is no previous report.

### Phylogenetic and Selection Analyses

We first reconstructed the phylogenies of the whole *Or* and *Gr* families, containing 3,545 and 806 sequences respectively, as following: 1) translated protein sequences were aligned using MAFFT v7.215 (Katoh and Standley 2013) with the high-accuracy option “E-INS-i”; 2) the alignment was filtered using trimAl v1.4 (Capella-Gutierrez et al. 2009) with the “automated1” option; 3) a maximum-likelihood (ML) tree was estimated using RAxML v8.1.16 (Stamatakis 2014) with the “PROTCATJTTF” model and 100 bootstrap (BS) replicates; 4) the protein sequences were aligned again using MAFFT (“E-INS-i”) with the ML tree as guide; 5) the alignment was evaluated using GUIDANCE v1.5 (Penn et al. 2010) with the 100 BS trees from step 3) and unreliably aligned residues (confidence score <0.8) were masked; 6) gap-rich columns were removed using trimAl with the “gappyout” option; and 7) the final ML tree was generated as in step 3).

Subfamilies were delineated from the whole family phylogenies and analyzed individually as following: 1) protein sequences were aligned using MAFFT (“E-INS-i”); 2) the alignment was evaluated using GUIDANCE with 100 BS replicates and residues with score <0.8 were masked; 3) gap-rich columns were removed using trimAl (“gappyout”); 4) a ML tree was estimated using RAxML with the “PROTGAMMAAUTO” model and 100 BS replicates; and 5) steps 1) to 4) were repeated, only that the alignment was performed using PRANK v140603 (Loytynoja and Goldman 2008) with the “-F” option and the ML tree as guide, and the 100 BS trees were used for the GUIDANCE evaluation. The final protein sequence alignments were reverse-translated into codon alignments for selection analysis.

**Fig. 1.—:**
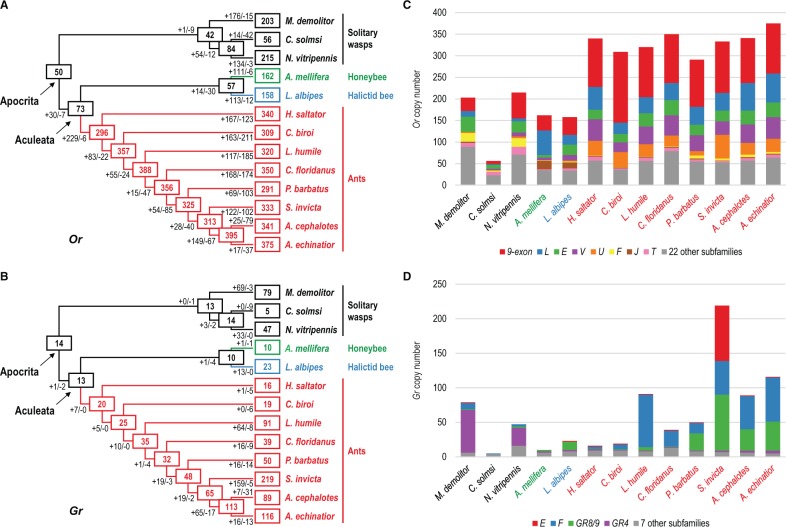
Copy number dynamics of chemoreceptors in Hymenoptera. (*A*, *B*) Numbers of *Or* (*A*) and *Gr* (*B*) genes in extant species, and estimated numbers of ancestral copies. Estimated numbers of gene gain and loss events are shown on each branch with plus and minus signs respectively. The relationships between the 13 hymenopteran species are from (Moreau, et al. 2006; Klopfstein, et al. 2013; Kocher, et al. 2013). (*C*, *D*) Numbers of *Or* (*C*) and *Gr* (*D*) genes in extant species breakdown by subfamily. Several large subfamilies showed differential expansion patterns between lineages (e.g. the *Or* subfamilies *U*, *J*, and *F* were specifically expanded in ants, bees, and wasps, respectively) and species of the same lineage (e.g. the size of the *Gr* subfamily *F* differed substantially between ants). Species names are colored according to their lineages: black – solitary wasps; green – honeybee; blue – halictid bee; red – ants.

To estimate the number of gene gain and loss events, the phylogenetic trees of subfamilies were first corrected using TreeFix v1.1.10 (Wu et al. 2013) with the “long” search settings given the reference organismal phylogeny shown in figure 1; the corrected trees were then reconciled with the same organismal phylogeny using NOTUNG v2.8 (Chen et al. 2000). All selection analyses were based on trees corrected using TreeFix as well. The results of *Or* and *Gr* subfamilies were then summed, respectively, to reflect the copy number dynamics of the whole families. The global ω ratio of each subfamily was estimated using HyPhy v2.2.4 (Pond et al. 2005). The recently developed adaptive branch-site random effects likelihood (aBSREL) approach (Smith et al. 2015) implemented in HyPhy was used to detect evidence of episodic positive selection on all branches in all subfamilies. Some very large subfamilies, including *Or* subfamilies *9-exon*, *E*, *L*, *V*, and *Gr* subfamily *F*, were condensed by removing selected ant and wasp species (supplementary table S5 and S6, Supplementary Material online) to make the analyses feasible. The FDR method (Benjamini and Hochberg 1995) was applied to the *P* values of all 5,439 tests of *Or* and *Gr* branches to correct for multiple testing.

### Gene Expression Analysis

The following antennal transcriptome data were obtained from their respective sources (supplementary table S9, Supplementary Material online): *A. mellifera* nurses and foragers (Jasper et al. 2015); *C. biroi* workers and males (McKenzie et al. 2014); *C. floridanus* major workers and males (Zhou et al. 2012); as well as *H. saltator* workers and males (Zhou et al. 2012). Reads were trimmed for low-quality positions and then aligned to respective genome assemblies using STAR v2.4.0 (Dobin et al. 2013) with gene annotations as guidance (supplementary table S9, Supplementary Material online). Uniquely mapped reads were counted using HTSeq v0.6.1p1 (Anders et al. 2015) and reads per kilobase per million mapped reads (RPKM) values were calculated for all genes as a measure of expression level. Percentile ranks of genes’ RPKM values in their respective transcriptomes were determined, and the ranks of *Or*s were compared between *A. mellifera* and each ant using the Wilcoxon rank-sum test. GFOLD v1.1.2 (Feng et al. 2012) was used to determine the fold changes in transcript abundances of genes between workers and males in ants. To account for potential bias introduced by using only uniquely mapped reads, Cuffdiff v2.2.1 (Trapnell et al. 2013) (which incorporates nonuniquely mapped reads as well) was used as an alternative approach to calculate the transcript abundances and fold changes and the results were highly similar (supplementary fig. S3 and table S10, Supplementary Material online).

### Data Availability

This project has been deposited at Figshare under the accession 10.6084/m9.figshare.1485695. The deposited data include all the sequences, raw and filtered alignments, phylogenetic trees of *Or* and *Gr* families and subfamilies, results of gene gain/loss and selection analyses, and commands for software tools used in this study.

## Results and Discussion

### Both Solitary and Social Hymenopterans Contain Large Chemoreceptor Repertoires

We annotated Or and Gr genes in two solitary wasps (*C. solmsi* and *M. demolitor*), one halictid bee (*L. albipes*), and five ants (*Acromyrmex echinatior*, *Atta cephalotes*, *Cardiocondyla obscurior*, *Monomorium pharaonis*, and *Solenopsis invicta*). As a result, a large number of chemoreceptor genes were identified in these species (table 1; supplementary table S1, Supplementary Material online). We then compared the size of all currently characterized chemoreceptor repertoires in Hymenoptera. To streamline downstream analyses, we focused in this study on nearly intact genes encoding proteins of 350 or more amino acids.

The numbers of *Or*s are largely comparable among ants where most species have 300 or more copies (fig. 1*A* and table 1). The tramp ant *C. obscurior* has notably fewer *Or*s than other ants. The genome of *C. obscurior* is the smallest among sequenced ants and contains genomic regions with high densities of transposable elements (TEs) in which *Or*s were enriched (Schrader et al. 2014), suggesting that *C. obscurior Or*s are likely to be affected by genome streamlining and random copy number changes caused by TE activity. There is a reduced number of *Or*s in the pharaoh ant *M. pharaonis* as well, however it is likely biased by the current, highly fragmented genome assembly. The two bees each has ∼160 *Or*s, highly consistent with the recent report of 159 *Or*s in bumblebee (Sadd et al. 2015). On the other hand, while both *N. vitripennis* and *M. demolitor* have ∼200 *Or*s, we found only 56 *Or*s in the fig wasp *C. solmsi* which is an obligate specialist on figs and may have likely experienced a reduction in its chemosensory gene repertoire (Xiao et al. 2013). More wasp genomes are needed to better characterize the variability of *Or* family size in this lineage.

In the *Gr* family, however, our results further strengthen the previous observation that *Gr* copy numbers vary considerably even between related species (fig. 1*B* and table1). For instance, three of the five ant genomes we annotated in this study have more than 100 *Gr*s, whereas most previously analyzed ants have 50 or less copies. Strikingly, we found 219 *Gr*s in the highly invasive fire ant *S. invicta*, on a par with the highest number of *Gr*s previously reported in insects (*Tribolium castaneum* has 220 intact *Gr*s) (Tribolium Genome Sequencing Consortium 2008). Interestingly, *M. pharaonis* which is also an invasive ant has a greatly expanded *Gr* family as well, despite its fragmented genome assembly. Among wasps, while *C. solmsi* has only five *Gr*s, which is consistent with its specialist lifestyle, *M. demolitor* contains almost twice as many *Gr*s as *N. vitripennis* (79 vs. 47). On the contrary, both *A. mellifera* and *L. albipes* have relatively small *Gr* repertoires, potentially reflecting the much reduced need for gustatory reception due to the mutualistic relationships between bees and plants (Robertson and Wanner 2006).

The vast majority of the chemoreceptor genes, we annotated here are tandemly arrayed in genomes (supplementary fig. S1, Supplementary Material online). Tandem duplication is known to have important contributions to the chemoreceptor repertories in most insect genomes (Robertson et al. 2003; Engsontia et al. 2008; Wanner and Robertson 2008; Smadja et al. 2009), particularly so in Hymenoptera (Robertson and Wanner 2006; Robertson et al. 2010; Smith, Zimin, et al. 2011; Smith, Smith, et al. 2011; Zhou et al. 2012; Sadd et al. 2015). Indeed, we found the largest known chemoreceptor gene clusters in the leafcutter ant *A. cephalotes* (a cluster of 68 *Or*s) and the fire ant (a cluster of 66 *Gr*s). The formation of tandem clusters may accelerate the turnover of chemoreceptors as one duplication (or loss) event can involve more than one gene simultaneously (see one such example in the bumblebee genome [Sadd et al. 2015]).

### Patterns of Chemoreceptor Expansions Are Highly Diverse between Subfamilies and between Species

Our phylogenetic analyses of the *Or* and *Gr* genes in 13 solitary and social hymenopterans (fig. 1) found 30 and 11 well-supported subfamilies (supplemental table S1, Supplementary Material online) respectively, each representing potentially one or a few genes in the common ancestor of the suborder Apocrita within Hymenoptera (wasps, bees, and ants). These include all previously reported subfamilies (Zhou et al. 2012) and also several additional subfamilies that were previously orphan lineages. We then reconstructed the histories of gain and loss for *Or* and *Gr* genes to examine the dynamics of chemoreceptor gene numbers in solitary and social hymenopterans (fig. 1*A* and *B*; supplementary table S2 and S3, Supplementary Material online). Consistent with our previous study (Zhou et al. 2012), the results showed that the respective ancestors of Apocrita, Aculeata (bees and ants), the three solitary wasps, and the two bees all had relatively small number of chemoreceptors, suggesting that the many chemoreceptors found in wasps and bees were mostly derived from lineage-specific expansions. Similarly, the ants with higher numbers of *Gr*s all showed clear patterns of recent duplications (fig. 1*B*). Our current analysis including additional early-branching ant species also confirmed the dramatic expansion of *Or*s inferred in the common ancestor of ants. Subsequent duplication/loss events occurred at high rates in all ant lineages despite their overall similar *Or* numbers (fig. 1*A*).

The vast majority of gene gains and losses can be attributed to a few large subfamilies which also displayed divergent evolutionary patterns between species (fig. 1*C* and *D*; supplementary fig. S2 and S3, Supplementary Material online). For instance, the *9-exon* subfamily is the largest *Or* subfamily in ants, halictid bee, and jewel wasp, containing at least 25% of the *Or*s in each of these species (fig. 1*C*). However, the honeybee has many more *Or*s in the subfamily *L*, and the other wasp *M. demolitor* has 50% less *9-exon Or*s compared with *N. vitripennis*. Even between ants, the number of *9-exon Or*s can differ dramatically (e.g. 104 in *A. cephalotes* vs. 164 in *C. biroi*). Additionally, ants, bees, and wasps each showed lineage-specific expansions in the subfamilies *U*, *J*, and *F*, respectively (fig. 1*C*). Similarly, while the majority of ant *Gr* duplications was harbored in the subfamilies *F* and *Gr8/9*, expansions of wasp *Gr*s occurred almost exclusively in the subfamily *Gr4* (fig. 1*D*). Particularly, a *S. invicta*-specific expansion included 79 *Gr*s in the subfamily *E* where all other species including ants have either none or only one copy (fig. 1*D*). In sum, our results revealed highly diverse patterns of gene gain and loss among subfamilies of chemoreceptors and among species.

### Chemoreceptors Have Experienced Elevated Positive Selection in Halictid Bee and the Ancestor of Ants

To investigate the sequence level evolution of chemoreceptors, we first examined the global selective pressure (the ratio of nonsynonymous/synonymous substitution rates, or ω) for all subfamilies and revealed a diverse range of ω values (supplementary table S4, Supplementary Material online). In both *Or* and *Gr* families, the strongest levels of purifying selection were found in subfamilies that are strictly single-copy in all species, namely *Orco* (ω = 0.044), which encodes the coreceptor required for Or function, and *Gr1/Gr2* (ω = 0.105 and 0.155), which likely encode sugar receptors (Robertson and Wanner 2006). Conversely, the highest ω values were displayed by the most expanded subfamilies in each family, including the *Or* subfamily *9-exon* (ω = 0.384), as well as the *Gr* subfamilies *E*, *F*, and *Gr8/9* (ω = 0.484, 0.500, and 0.474, respectively), suggesting increased levels of functional divergence in these subfamilies due to much relaxed selection.

We then performed branch-site tests (see Materials and Methods) to detect episodic positive selection during chemoreceptor gene evolution in Hymenoptera. We quantified the respective proportions of branches under positive selection in wasps, halictid bee, honeybee, and ants, representing one solitary lineage and three independent origins of eusociality (table 2; supplementary tables S5 and S6, Supplementary Material online). Notably, halictid bee branches showed evidence of positive selection much more frequently in both *Or* and *Gr* families. The evolution of sociality in the halictid bee family is highly dynamic; there are multiple recent origins of eusociality, and social polymorphisms are often displayed by closely related species or even populations of the same species (e.g. in *L. albipes*) (Brady et al. 2006). As such, halictid bees can be viewed to be at a relatively early stage of eusocial evolution. Our results suggest that chemoreceptors may have played an adaptive role in the establishment of eusociality in this lineage. Interestingly, one of the six genes under accelerated evolution between social and solitary *L. albipes* individuals is an *Or* (Kocher et al. 2013).

**Table 2:** Numbers of *Or* and *Gr* Branches under Episodic Positive Selection in Solitary and Social Hymenopteran Lineages

Lineage^a^		*Or*			*Gr*		
		Total Tested	*P* ≤ 0.05	FDR ≤ 0.10	Total Tested	*P* ≤ 0.05	FDR ≤ 0.10
Solitary wasps		796	125 (15.70%)	46 (5.78%)	237	27 (11.39%)	7 (2.95%)
Halictid bee		270	77 (28.52%^b^)	37 (13.70%^b^)	36	15 (41.67%^b^)	7 (19.44%^b^)
Honeybee		272	29 (10.66%^b^)	11 (4.04%)	11	2 (18.18%)	0 (0%)
Ants	all	2,894	329 (11.37%^b^)	95 (3.28%^b^)	1,135	191 (16.83%^b^)	70 (6.17%)
	ancestral	261	58 (22.22%^b^)	19 (7.28%)	17	2 (11.76%)	1 (5.88%)

^a^Branches leading exclusively to genes in the three wasps, *L. albipes*, *Apis mellifera*, and the eight ants were respectively classified as “solitary wasps,” “halictid bee,” “honeybee,” and “ants.” Branches corresponding to the node “n22” in the organismal phylogeny shown in supplementary table S2, Supplementary Material online were classified as “ancestral.”

^b^Significantly different from solitary wasps (*P* < 0.05; Fisher’s exact test).

However, overall, ant *Or*s showed lower level of positive selection relative to solitary wasps (table 2), consistent with previous findings comparing the jewel wasp and two of the ants (Roux et al. 2014). In light of the predicted dramatic expansion of *Or*s in the ant common ancestor and the aforementioned results in *L. albipes*, we further characterized the pattern of adaptive evolution on branches representing the ant common ancestor (i.e. branches in gene phylogenies that correspond to the branch leading to the node “n22” in the organismal phylogeny shown in supplementary table S2, Supplementary Material online). Indeed, we found elevated level of positive selection on *Or*s during early ant evolution compared with the overall levels in ants or wasps (table 2; although not significant at 10% FDR level in the latter case). On the other hand, ant *Gr*s showed positive selection mostly among recent duplicates, likely reflecting a greater role for *Gr*s in species-specific adaptations.

Honeybee chemoreceptors similarly displayed lower overall levels of positive selection than wasps (table 2). It is possible that the use of other communication mechanisms such as the dance language (Seeley 1995) in honeybee reduced the adaptive pressure on chemoreceptors. Alternatively, chemoreceptors may have experienced greater levels of positive selection at particular stages in the evolution of honeybees, however the detection of such episodic events is difficult given that *A. mellifera* is the only species sampled in the family Apidae. Indeed, a recent population genomic study comparing four genetically distinct *A. mellifera* populations found *Or*s to be enriched among genes under positive selection during recent evolution of honeybees (Harpur et al. 2014). At larger scale, the origin of eusociality and transition from simple to complex eusociality occurred multiple times in Apidae (Woodard et al. 2011). Future analyses including more solitary and social members of Apidae will be necessary to better understand the evolution of chemoreceptors in this family.

### *Ors* Showed Divergent Expression Patterns between Honeybee and Ants

**Fig. 2.—:**
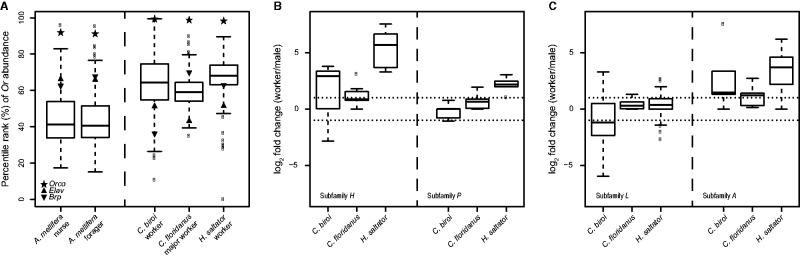
Divergent *Or* expression patterns between honeybee and ants. (*A*) The percentile ranks of *Or* transcript abundances in the antennal transcriptomes of *Apis mellifera* nurse and forager, as well as the workers of *Cerapachys biroi*, *Camponotus floridanus*, and *Harpegnathos saltator*. The star symbol indicates *Orco*. The percentile ranks of two neuronal marker genes, *Elav* (triangle symbol) and *Brp* (reverse triangle symbol), are shown for comparison. (*B*) Antennal expression patterns of ant *Or*s in subfamilies *H* and *P* where certain honeybee members showed worker-enrichment. (*C*) Antennal expression patterns of ant *Or*s in subfamilies *L* and *A* where certain honeybee members showed male-enrichment. The dotted lines indicate log2 fold changes of 1 and −1.

The recent availability of antennal transcriptome data from honeybee (Jasper et al. 2015) and several ants (Zhou et al. 2012; McKenzie et al. 2014) allows for a comparison of *Or* transcript abundance profiles between social lineages. We used the percentile rank of each *Or*’s transcript abundance for the cross-species comparison to account for differences in sample preparation, sequencing methodologies, and species. Interestingly, we found much higher overall *Or* abundances in the workers of all three ants relative to both young and old *A. mellifera* workers (median percentile rank: 59.0–68.1% vs. 40.7–41.3%; Wilcoxon rank-sum test *P* value < 1e-15 for all pairwise comparisons between ant and honeybee workers), while the two neuronal markers (*Elav* and *Brp*) displayed similar or lower abundances in ants compared with *A. mellifera* (fig. 2*A*; supplementary table S7, Supplementary Material online). The elevated *Or* abundances in worker antennae of ants, together with their substantially larger chemoreceptor repertoires, suggest a higher chemosensory ability of ant workers which is concordant with the more complex ant chemical communication systems. Additionally, in contrast to ants and other insects such as mosquitoes (Pitts et al. 2011; Zhou et al. 2014), the obligate *Orco* coreceptor is not the most abundant *Or* in *A. mellifera* worker antenna (fig. 2*A*), further indicating divergent *Or* expression between honeybee and ants.

We further compared the sex-specific patterns of honeybee and ant *Or*s. A previous study based on microarray and quantitative-PCR has identified several worker-/male-biased *A. mellifera Or*s (*AmOr*s) (Wanner et al. 2007), and examination of ant *Or*s in the same subfamilies revealed both conserved and divergent patterns (fig. 2*B* and *C*; supplementary table S7, Supplementary Material online). Many ant *Or*s in the subfamilies *H* and *P* showed worker-enrichment, consistent with *AmOr150/Or151* and *AmOr63* in the respective clades (fig. 2*B*). Importantly, several *AmOr*s in the subfamily *L* were more abundant in males, including *AmOr11* which encodes a receptor for (*E*)-9-oxo-2-decenoic acid (9-ODA), a major queen substance that functions in both attracting males and regulating worker reproduction (Wanner et al. 2007). Although the use of 9-ODA seems highly specific to *A. mellifera* (Van Oystaeyen et al. 2014), a number of *H. saltator* and *C. biroi Or*s in this subfamily also showed strong enrichment in males (fig. 2*C*), suggesting their potential function as sex pheromone receptors as well. The lack of male-enriched subfamily *L Or*s in *Camponotus floridanus*, on the other hand, may indicate an alteration in its use and/or perception of sex pheromone (fig. 2*C*). Interestingly, unlike *AmOr170*, ant *Or*s in the subfamily *A* were mostly enriched in workers (fig. 2*C*), indicating a functional divergence between the two lineages.

### Implications for the Evolution of Sociality

In this study, we have characterized a large number of *Or* and *Gr* genes in eight hymenopteran genomes; the annotations themselves are likely to be valuable resources for future comparative and functional studies. More importantly, the analyses of these chemoreceptors provide insights into their evolution and their roles in the evolution of social Hymenoptera. Our phylogenetic comparison of multiple solitary and social lineages indicates that an expanded chemoreceptor repertoire is likely a general feature of Apocrita without any exclusive association with sociality (Fischman et al. 2011). At the same time, all social insects sequenced so far have complex chemical communication systems and moreover contain relatively large chemoreceptor repertoires (note that the damp-wood termite harbors a greatly expanded family of *Ir*s instead of *Or*s and *Gr*s [Terrapon et al. 2014]). The net gain of more than 200 *Or*s in the ant common ancestor also represents one of the most striking expansions of insect chemoreceptors. Furthermore, we found elevated positive selection on chemoreceptors during the transition to eusociality in two independent lineages (halictid bees and ants), consistent with an adaptive role of chemoreceptors in social evolution.

These findings raise the possibility that the frequent expansion of chemoreceptors, while allowing for rapid adaptation to changing chemical environments, also independently increase the probability of developing sophisticated chemical communication mechanisms compatible with the emergence of social life. For example, nestmate and caste recognition in social insects are largely mediated by the CHC profiles whose composition can be highly complex and vary qualitatively and quantitatively between species, colonies, and castes (Blomquist and Bagneres 2010). Functional divergence between duplicated chemoreceptors may allow them each to be tuned for a subset of CHCs, thus collectively achieving a high specificity and accuracy in communication. Furthermore, as illustrated by the divergent patterns of gene expansion and expression revealed in our analyses, independently evolved social lineages often explore different subsets of chemoreceptors for their communication. This observation again highlights the possibility that the numerous chemoreceptor duplications provide the potential for complex social communications but the details of how these duplications have contributed to eusociality are likely to have varied in each case. Recent genome-wide comparisons among multiple ants (Simola et al. 2013) and bees (Kapheim et al. 2015) revealed a similar trend that independent origins of eusociality differ considerably in their underlying molecular details (Johnson and Linksvayer 2010; Johnson and Tsutsui 2011).

Finally, our results suggest several future directions. First, functional information about chemoreceptors is critical to linking the evolution and expression patterns of these genes with the ecology of organisms. Such data are extremely scarce in Hymenoptera and there is an urgent need for relevant functional characterizations. Second, given that honeybee chemoreceptors displayed the lowest levels of episodic positive selection, it would be interesting to further investigate the patterns of selection in the very recently sequenced genomes of ten bees that encapsulate a range of social complexity (Kapheim et al. 2015). Lastly, as our results suggest a potential, adaptive role of chemoreceptors in the transition to eusociality, future studies comparing closely related species (or populations of the same species) that are socially polymorphic may provide further insights. Similarly, the comparison of populations exhibiting alternative social organizations (e.g. monogyne and polygyne forms of *S. invicta*) may help to elucidate the role of chemoreceptors in the further development of eusociality. 

## Supplementary Material

Supplementary figures S1–S3 and tables S1–S10 are available at *Genome Biology and Evolution* online (http://www.gbe.oxfordjournals.org/).

Supplementary Data
