# Supplementary material for: Chemoreceptor Evolution in Hymenoptera and Its Implications for the Evolution of Eusociality
Source: Genome Biol Evol. 2015 Aug 12;7(8):2407–16. doi: 10.1093/gbe/evv149 (PMC4558866; doi:10.1093/gbe/evv149)
Supplement: Supplementary Data [file supp_7_8_2407__index.html]

Chemoreceptor evolution in Hymenoptera and its implications for the evolution of eusociality — Chemoreceptor Evolution in Hymenoptera and Its Implications for the Evolution of Eusociality — Supplementary Data 

# Chemoreceptor Evolution in Hymenoptera and Its Implications for the Evolution of Eusociality

## Supplementary Data

files

- Supplementary Data - zip file
