# Supplementary material for: Chemoreceptor Evolution in Hymenoptera and Its Implications for the Evolution of Eusociality
Source: Genome Biol Evol. 2015 Aug 12;7(8):2407–16. doi: 10.1093/gbe/evv149 (PMC4558866; doi:10.1093/gbe/evv149)
Supplement: Supplementary Data [file supp_evv149_suppl_data.zip › Supplementary figures.pdf]

Supplementary Figure Legend

Figure S1: Tandem duplication has major contribution to the chemoreceptor repertoires of most insects. Scatter plots of *Or* (A) and *Gr* (B) gene numbers in 22 insect genomes versus the fractions of tandemly arrayed genes. The plots include data from the following insect genomes (sources of chemoreceptor gene annotations are indicated in parenthesis): **Coleoptera** - *Tribolium castaneum* (Engsontia, et al. 2008); **Diptera** - *Anopheles gambiae* (Zhou, et al. 2014), *Aedes aegypti* (Zhou, et al. 2014), *Bombyx mori* (annotated in this study using genome assembly version 2 from SilkDB), *Culex quinquefasciatus* (Zhou, et al. 2014), *Drosophila melanogaster* (Gardiner, et al. 2008); **Hemiptera** - *Acyrthosiphon pisum* (Smadja, et al. 2009); **Hymenoptera** - *Acromyrmex echinatior* (annotated in this study), *Apis mellifera* (Robertson and Wanner 2006), *Atta cephalotes* (annotated in this study), *Camponotus floridanus* (Zhou, et al. 2012), *Cardiocondyla obscurior* (annotated in this study), *Cerapachys biroi* (Oxley, et al. 2014), *Ceratosolen solmsi* (annotated in this study), *Harpegnathos saltator* (Zhou, et al. 2012), *Lasioglossum albipes* (annotated in this study), *Linepithema humile* (Smith, Zimin, et al. 2011), *Microplitis demolitor* (annotated in this study), *Monomorium pharaonis* (annotated in this study), *Nasonia vitripennis* (Robertson, et al. 2010), *Pogonomyrmex barbatus* (Smith, Smith, et al. 2011), *Solenopsis invicta* (annotated in this study); **Lepidoptera** - *Danaus plexippus* (annotated in this study using genome assembly version 3 from MonarchBase). In this study, *Or* or *Gr* genes that are less than 50 kibobase apart are considered tandem arrayed duplicates.

Figure S2: A few large subfamilies contribute the majority of chemoreceptor gene gain and loss events in Hymenoptera. *Or* (A) and *Gr* (B) gene gain and loss events breakdown by subfamily.

Figure S3: Divergent *Or* expression patterns between honeybee and ants. The results shown in this figure are generated by Cuffdiff analyses, while those in Figure 2 are generated by HTSeq/GFOLD analyses. (A) The percentile ranks of *Or* transcript abundances in the antennal transcriptomes of *Apis mellifera* nurse and forager, as well as the workers of *Cerapachys biroi, Camponotus floridanus*, and *H. saltator*. The star symbol indicates *Orco*. The percentile ranks of two neuronal marker genes, *Elav* (triangle symbol) and *Brp* (reverse triangle symbol), are shown for comparison. (B) Antennal expression patterns of ant *Or*s in subfamilies *H* and *P* where certain honeybee members showed worker-enrichment. (C) Antennal expression patterns of ant

*Or*s in subfamilies *L* and *A* where certain honeybee members showed male-enrichment. The dotted lines indicate log2 fold changes of 1 and -1.

References

Engsontia P, Sanderson AP, Cobb M, Walden KK, Robertson HM, Brown S. 2008. The red flour beetle's large nose: an expanded odorant receptor gene family in Tribolium castaneum. *Insect Biochem Mol Biol*. 38:387-397.

Gardiner A, Barker D, Butlin RK, Jordan WC, Ritchie MG. 2008. Drosophila chemoreceptor gene evolution: selection, specialization and genome size. *Mol Ecol*. 17:1648-1657.

Oxley PR, Ji L, Fetter-Pruneda I, McKenzie SK, Li C, Hu H, Zhang G, Kronauer DJ. 2014. The genome of the clonal raider ant Cerapachys biroi. *Curr Biol*. 24:451-458.

Robertson HM, Gadau J, Wanner KW. 2010. The insect chemoreceptor superfamily of the parasitoid jewel wasp Nasonia vitripennis. *Insect Mol Biol*. 19 Suppl 1:121-136.

Robertson HM, Wanner KW. 2006. The chemoreceptor superfamily in the honey bee, Apis mellifera: expansion of the odorant, but not gustatory, receptor family. *Genome Res*. 16:1395-1403.

Smadja C, Shi P, Butlin RK, Robertson HM. 2009. Large gene family expansions and adaptive evolution for odorant and gustatory receptors in the pea aphid, Acyrthosiphon pisum. *Mol Biol Evol*. 26:2073-2086.

Smith CD, Zimin A, Holt C, Abouheif E, Benton R, Cash E, Croset V, Currie CR, Elhaik E, Elsik CG, et al. 2011. Draft genome of the globally widespread and invasive Argentine ant (Linepithema humile). *Proc Natl Acad Sci U S A*. 108:5673-5678.

Smith CR, Smith CD, Robertson HM, Helmkampf M, Zimin A, Yandell M, Holt C, Hu H, Abouheif E, Benton R, et al. 2011. Draft genome of the red harvester ant Pogonomyrmex barbatus. *Proc Natl Acad Sci U S A*. 108:5667-5672.

Zhou X, Rinker DC, Pitts RJ, Rokas A, Zwiebel LJ. 2014. Divergent and conserved elements comprise the chemoreceptive repertoire of the nonblood-feeding mosquito Toxorhynchites amboinensis. *Genome Biol Evol*. 6:2883-2896.

Zhou X, Slone JD, Rokas A, Berger SL, Liebig J, Ray A, Reinberg D, Zwiebel LJ. 2012. Phylogenetic and transcriptomic analysis of chemosensory receptors in a pair of divergent ant species reveals sex-specific signatures of odor coding. *PLoS Genet*. 8:e1002930.

Figure S1

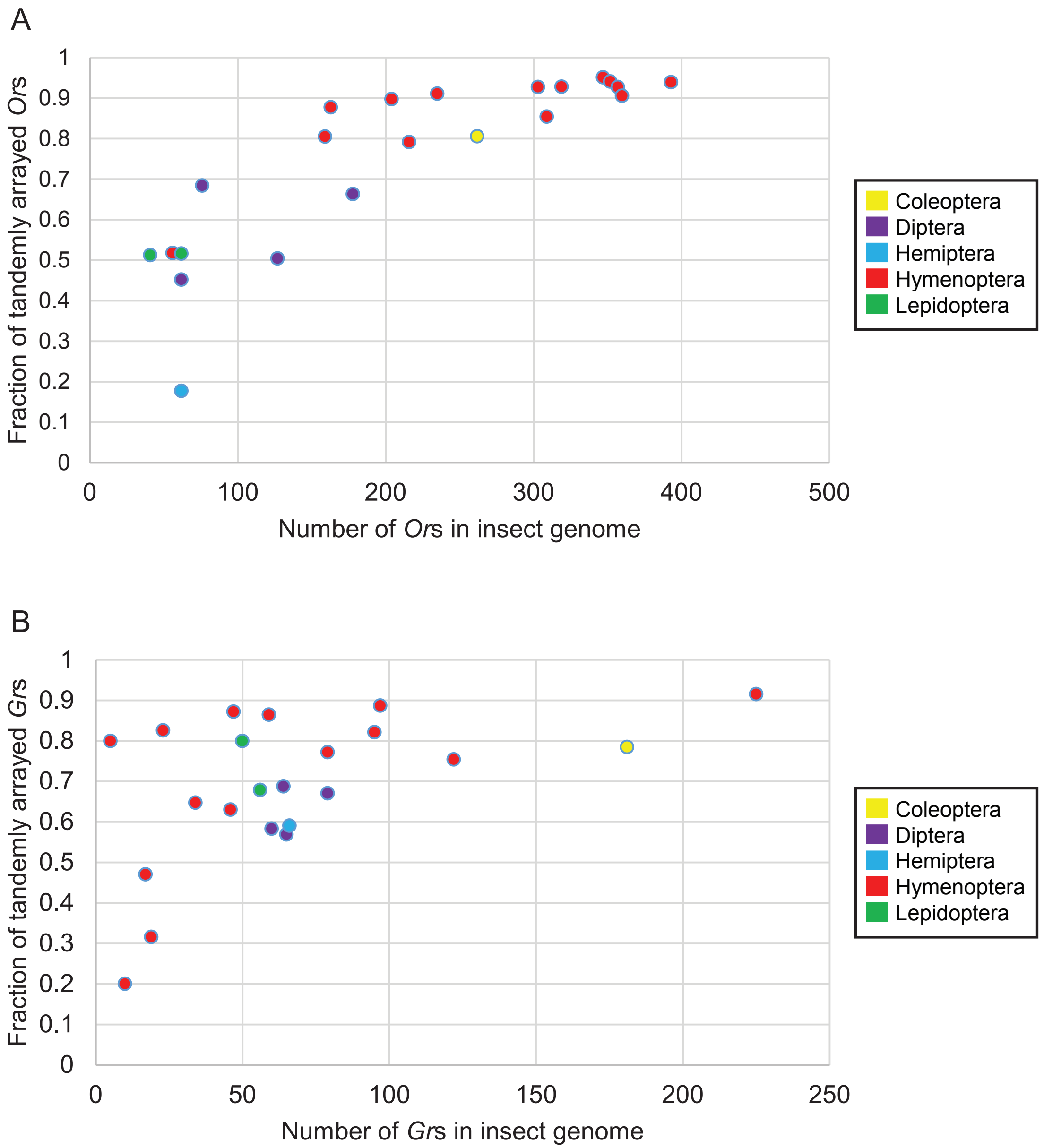
A
Fraction of tandemly arrayed *Or*s
Number of *Or*s in insect genome
0
0.1
0.2
0.3
0.4
0.5
0.6
0.7
0.8
0.9
1
0
100
200
300
400
500
Coleoptera
Diptera
Hemiptera
Hymenoptera
Lepidoptera
B
Fraction of tandemly arrayed *Gr*s
Number of *Gr*s in insect genome
0
50
100
150
200
250
Coleoptera
Diptera
Hemiptera
Hymenoptera
Lepidoptera

Figure S2

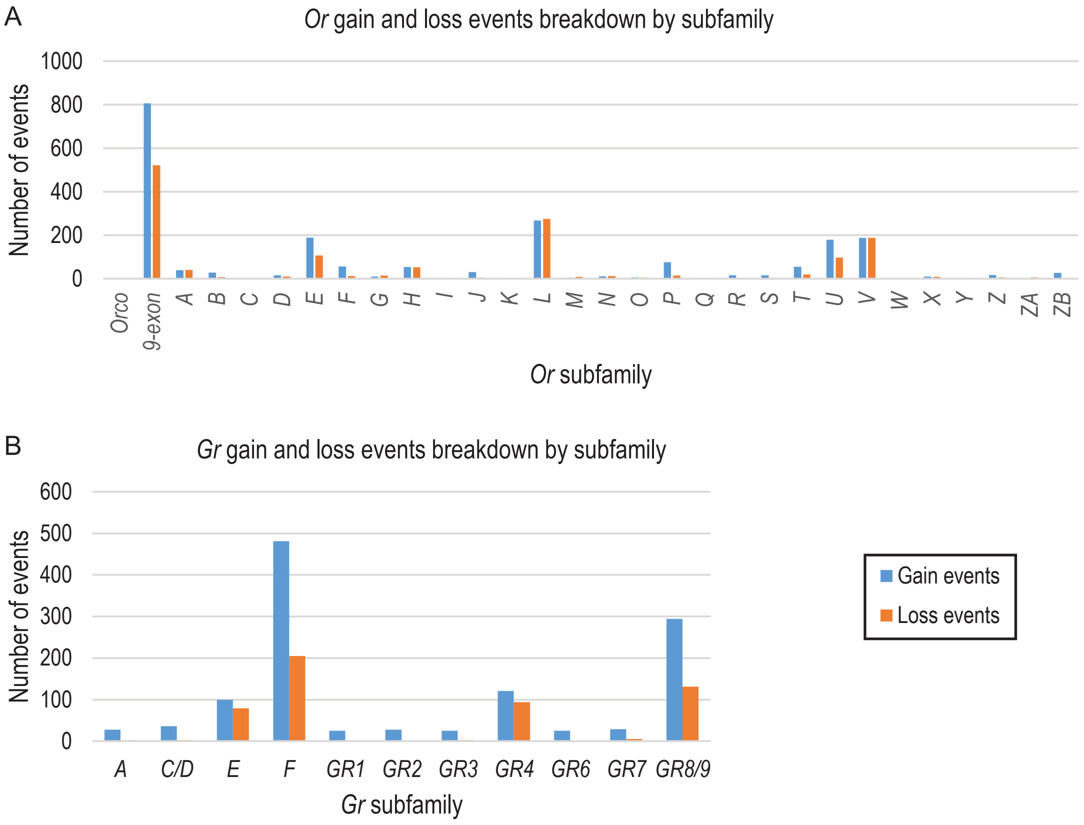
A
Or gain and loss events breakdown by subfamily
Number of events
1000
800
600
400
200
0
Orco
9-exon
A
B
C
D
E
F
G
H
I
J
K
L
M
N
O
P
Q
R
S
T
U
V
W
X
Y
Z
ZA
ZB
Or subfamily
B
Gr gain and loss events breakdown by subfamily
Number of events
600
500
400
300
200
100
0
A
C/D
E
F
GR1
GR2
GR3
GR4
GR6
GR7
GR8/9
Gr subfamily
Gain events
Loss events

Figure S3

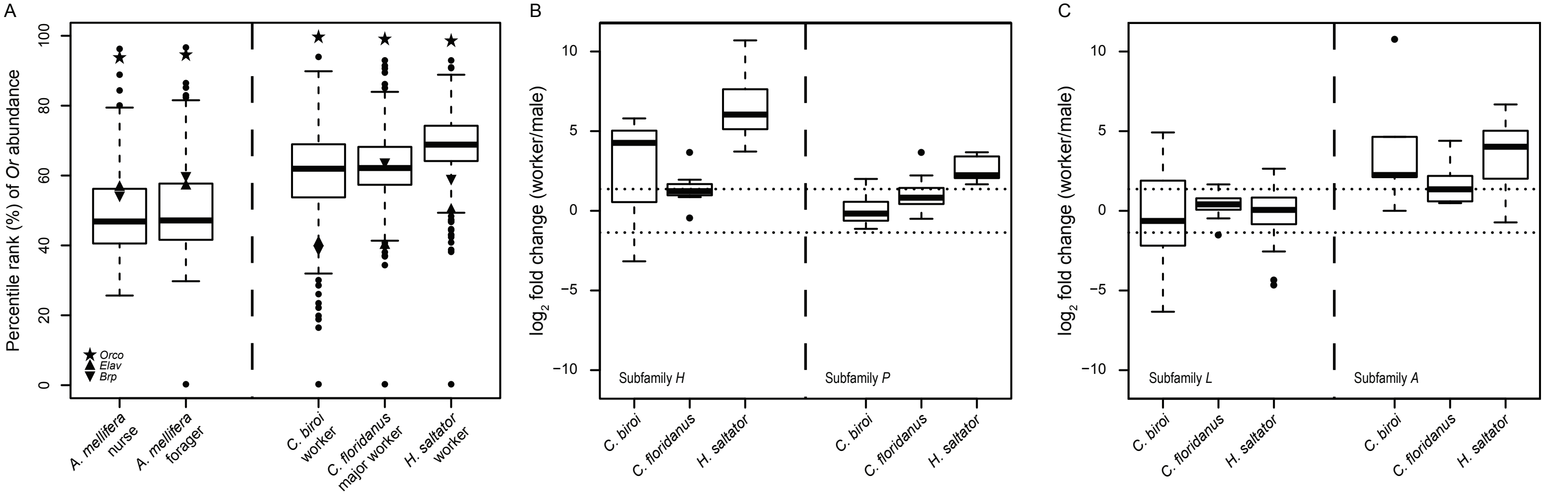
A
Percentile rank (%) of Or abundance
0
20
40
60
80
100
Orco
Elav
Brp
A. mellifera nurse
A. mellifera forager
C. biroi worker
C. floridanus major worker
H. saltator worker
B
log2 fold change (worker/male)
−10
−5
0
5
10
Subfamily H
Subfamily P
C. biroi
C. floridanus
H. saltator
C. biroi
C. floridanus
H. saltator
C
log2 fold change (worker/male)
−10
−5
0
5
10
Subfamily L
Subfamily A
C. biroi
C. floridanus
H. saltator
C. biroi
C. floridanus
H. saltator
